# Carbapenems: The Final Line of Defense in Typhoid Fever Treatment at Hayatabad Medical Complex, Peshawar

**DOI:** 10.7759/cureus.77855

**Published:** 2025-01-22

**Authors:** Khalid Shahab, Ahmad Zuhayr, Fatima Rizwan, Maria Noori, Laiba Bukhari, Sabina Shahid Waziry, Khawla Saeed, Hooria Malik, Hadia Khan

**Affiliations:** 1 Medicine, Khyber Girls Medical College, Peshawar, PAK; 2 Internal Medicine, Hayatabad Medical Complex Peshawar, Peshawar, PAK

**Keywords:** carbapenems, extensively drug-resistant, multidrug resistance, sensitivity, typhoid fever

## Abstract

Introduction: Typhoid fever, caused by *Salmonella enterica* serovar Typhi, remains a significant public health issue, particularly in low-income countries with inadequate sanitation. The rise of extensively drug-resistant (XDR) strains of *Salmonella* Typhi has led to a growing reliance on carbapenems as the final line of defense. This study aims to evaluate the effectiveness of carbapenems in treating multidrug-resistant (MDR) and XDR typhoid at Hayatabad Medical Complex, Peshawar.

Objective: This study aims to assess the efficacy of carbapenems in treating MDR and XDR typhoid fever and identify alternative therapeutic strategies.

Methodology: A retrospective cohort study was conducted at Hayatabad Medical Complex from January to June 2024. A total of 501 patients with confirmed typhoid fever were included. Data on demographics, drug sensitivity, and resistance patterns were extracted from the hospital's Health Management Information System (HMIS) and analyzed using IBM SPSS Statistics for Windows, Version 25 (Released 2017; IBM Corp., Armonk, New York). Statistical significance was set at p < 0.05.

Results: Among 501 patients, 299 (59.7%) were male, with a mean age of 23.84 ± 0.24 years. MDR was observed in 106 (36%) patients, while XDR and carbapenem resistance were present in only three (0.6%) cases. Imipenem demonstrated a high sensitivity of 466 (93%), while meropenem had 380 (75.8%) sensitivity with no resistance. Non-carbapenem antibiotics, such as polymyxin 468 (93.4%) sensitivity and colistin 446 (89%) sensitivity, exhibited higher efficacy than carbapenems. Alarmingly, cephalosporins such as cefepime exhibited 454 (91%) resistance. Resistance to carbapenems was rare, affecting only three (0.6%) patients.

Conclusion: Carbapenems remain highly effective against MDR and XDR typhoid, but non-carbapenem options such as polymyxin and colistin offer viable alternatives in reducing carbapenem dependence. Enhanced antimicrobial stewardship is critical to preserving these last-resort treatments.

## Introduction

Typhoid fever, caused by *Salmonella enterica* serovar Typhi, remains a critical global public health challenge, particularly in developing nations having regions with inadequate sanitation infrastructure. This disease is transmitted through contaminated food and water and affects an estimated 11-21 million individuals globally each year, with approximately 148,000-161,000 associated deaths [1,2]. The epidemiology of typhoid fever demonstrates significant regional variation, with some of the highest incidence rates reported in sub-Saharan Africa, notably in the Democratic Republic of the Congo, where the incidence exceeds 300 cases per 100,000 person-years [3]. In many low-income settings, typhoid fever disproportionately affects children and adults, with mortality rates ranging between 1% and 4% [4].

Despite advancements, challenges such as delayed diagnosis, rising antibiotic resistance, and limited vaccination coverage in endemic regions continue to hinder control efforts. Current treatment options include ceftriaxone and azithromycin, although resistance to fluoroquinolones remains a growing concern [5]. The World Health Organization (WHO) recommends the use of typhoid conjugate vaccines in high-incidence areas, yet coverage remains insufficient in many endemic regions [3].

The epidemiology of typhoid fever in Pakistan presents a concerning trend, particularly with the rise of extensively drug-resistant (XDR) strains, posing a significant public health challenge, especially in rural areas and among children. In Peshawar, a study reported a 12% prevalence of confirmed cases, with higher rates in children (15.6%) and males (13.8%) [6]. Between 2016 and 2020, over 22,000 cases were documented, with 15,717 identified as XDR [7]. XDR strains, which have been prevalent since 2016, account for 71% of pediatric isolates, further complicating treatment options as resistance to ceftriaxone and other antibiotics rises [8,9]. While the COVID-19 pandemic initially reduced typhoid cases due to improved sanitation, XDR strains persisted and surged post-pandemic [9]. The persistent threat of XDR strains highlights the need for urgent public health interventions and enhanced surveillance to address this growing crisis.

Recently, a study conducted by Imran et al. in Lahore, Pakistan, highlighted that 14.7% of cases are multidrug-resistant (MDR), while 43.4% exhibit XDR, with a concerning increase in resistance genes over three years [10]. Another study conducted by Al Masum et al. in Bangladesh reported that resistance to commonly used antibiotics, such as ampicillin and nalidixic acid, has reached 98% and 76%, respectively, while newer antibiotics, such as cefepime and meropenem, maintain 100% sensitivity [11]. Children, especially older age groups, are particularly vulnerable to XDR strains, necessitating combination therapies for effective treatment [12]. Current treatment strategies for XDR cases involve the use of carbapenems and macrolides, which have shown efficacy [11]. Alongside medical interventions, vaccination programs and public health education are critical in controlling the spread of resistant strains [13]. However, the ongoing emergence of resistance underscores the need for enhanced antimicrobial stewardship and comprehensive public health efforts to mitigate the impact of typhoid fever [13].

Carbapenems have emerged as a vital treatment option for XDR typhoid fever, particularly in regions with widespread antimicrobial resistance. The rising prevalence of MDR and XDR *Salmonella* Typhi underscores the necessity of preserving carbapenems for severe cases, as they remain among the few effective antibiotics against these resistant strains [10]. Resistance to first-line antibiotics, such as fluoroquinolones and third-generation cephalosporins, has led to a critical reliance on carbapenems and azithromycin for treating XDR infections [14]. While azithromycin and carbapenems remain the primary treatment recommendations for XDR cases, alternative approaches, such as oral carbapenems, are currently under investigation [15]. In addition, case reports highlight the potentially fatal complications associated with XDR typhoid, further reinforcing the importance of timely, targeted antibiotic therapy [14]. However, despite the essential role carbapenems play, the increasing threat of resistance to these drugs calls for stringent antimicrobial stewardship and ongoing exploration of alternative therapeutic options to mitigate the growing risk of untreatable typhoid infections [16].

The increasing prevalence of MDR and XDR strains of *Salmonella* Typhi has rendered many traditional antibiotics ineffective, including fluoroquinolones and third-generation cephalosporins. As a result, carbapenems have emerged as one of the last effective lines of treatment for XDR infections. However, the growing dependence on carbapenems poses a serious concern for antibiotic stewardship, given the threat of potential resistance.

This study aims to evaluate the effectiveness of carbapenems in treating MDR and XDR typhoid fever at Hayatabad Medical Complex, Peshawar. By analyzing clinical data and drug sensitivity patterns, the study seeks to address the misconception that carbapenems are the definitive typhoid treatment and explore alternative therapeutic options. The findings will contribute to the development of more balanced, evidence-based treatment strategies, aiming to reduce the overreliance on carbapenems while managing antimicrobial resistance in typhoid fever.

## Materials and methods

This retrospective cohort study was conducted at the outpatient medical department and emergency department of MTI-Hayatabad Medical Complex, Peshawar, Pakistan, over a six-month period from January 2024 to June 2024. Ethical approval (no. 2287) was obtained from the Institutional Review Board. The sample size was determined to be 504 patients, all of whom were diagnosed with typhoid fever based on blood culture reports. Patients of any age and gender admitted to the regular medical ward or seen in the OPD or emergency department during the study period were included, while those admitted to the high dependency unit (HDU) and medical ICU were excluded to ensure uniformity in clinical presentation and treatment context.

Patient data were retrieved from the hospital's Health Management Information System (HMIS). The blood culture reports of patients admitted during the study period were accessed, and relevant clinical data, including demographic details (age, gender, residency), admission type, drug sensitivity patterns, and resistance profiles, were extracted. All data were anonymized, ensuring no personally identifiable information was disclosed during the analysis or presentation of results.

A detailed pro forma was used to systematically record each patient's information, including blood culture results, antimicrobial susceptibility, and any patterns of resistance observed. Data regarding the efficacy of carbapenems were of particular interest, especially in cases where conventional antibiotics failed to treat MDR and XDR *Salmonella* Typhi.

MDR typhoid fever refers to cases where *Salmonella* Typhi is resistant to at least two first-line drugs, such as ampicillin, chloramphenicol, and co-trimoxazole. XDR typhoid fever is defined as cases where *Salmonella* Typhi remains susceptible to only two or fewer antimicrobial categories, typically displaying resistance to first-line drugs such as fluoroquinolones and third-generation cephalosporins, necessitating treatment with carbapenems.

Data were analyzed using IBM SPSS Statistics for Windows, Version 25 (Released 2017; IBM Corp., Armonk, New York). The mean ± standard deviation was calculated for numerical variables such as age. Frequencies and percentages were computed for categorical variables, including gender, residency, admission type, drug sensitivity patterns, and resistance to different antibiotics, MDR, and XDR. The chi-square test was employed to assess the relationship between various predictors, such as age, gender with drug resistance patterns, MDR, XDR, and treatment with carbapenems, keeping a p-value of < 0.05 considered statistically significant.

## Results

A total of 501 typhoid-positive patients were included in this study, conducted between January 2024 and June 2024, all of whom had complete electronic records. Among these patients, 299 (60%) were male, and 202 (40%) were female. The average age of the patients was 23.84 ± 0.24 years, with a minimum age of 15 years and a maximum age of 47 years. The geographical distribution of the patients indicates that the majority resided in Peshawar Cantt, Peshawar City, Swabi, and Swat, each contributing 8.4% of the total sample, corresponding to 42 of the total sample. Other notable areas included Buner (33, 6.6%), Charsadda (39, 7.8%), Hayatabad (28, 5.6%), and Malakand (39, 7.8%), reflecting a diverse regional representation. The baseline characteristics of the study population are summarized in Table 1.

**Table 1:** Demographic characteristics of the patients OPD: outpatient department

Baseline Characteristics		Frequency (Percentage, %)
Age	11 to 20 years	114 (22.8%)
	21 to 30 years	360 (71.9%)
	31 to 40 years	18 (3.6%)
	41 to 50 years	9 (1.8%)
Gender	Male	299 (59.7%)
	Female	202 (40.3%)
Mode of admission	OPD	236 (47.1%)
	Emergency	265 (52.9%)

Table 2 presents an analysis of the antibiotic sensitivity and resistance patterns of *Salmonella* Typhi, revealing varying effectiveness across different antibiotics. Notably, imipenem exhibited a remarkable 93% sensitivity with only 0.6% resistance, while meropenem demonstrated a sensitivity of 75.8% with no reported resistance, underscoring their efficacy as treatment options. However, certain non-carbapenem antibiotics, such as polymyxin, displayed even greater effectiveness, achieving 93.4% sensitivity with a minimal resistance rate of 2.6%. Colistin also showed strong performance, with 89% sensitivity and 3.2% resistance. Additional antibiotics, such as tigecycline, offered 78.4% sensitivity but had a resistance rate of 14.8%, making it a viable option, albeit less effective than polymyxin and colistin. Amikacin and ciprofloxacin exhibited moderate effectiveness with sensitivities of 67.1% and 71.5%, respectively, but demonstrated higher resistance rates compared to carbapenems and colistin. Conversely, certain cephalosporins, including cefepime and ceftazidime, showed alarmingly high resistance levels, with 91% and 67% resistance, respectively, which severely limits their therapeutic applicability. In summary, while carbapenems such as imipenem and meropenem remain highly effective, non-carbapenems such as polymyxin and colistin offer superior results, characterized by high sensitivity and minimal resistance, thereby presenting strong alternatives in the treatment of *Salmonella* Typhi.

**Table 2:** Antibiotic resistance and sensitivity pattern of Salmonella Typhi

Antibiotic	Resistance	Sensitivity
Amikacin	152 (30.3%)	336 (67.1%)
Amoxicillin	361 (72%)	92 (18.4%)
Ampicillin	24 (4.8%)	1 (0.2%)
Azithromycin	134 (26.7%)	341 (68%)
Ceftazidime	335 (67%)	123 (25%)
Cefotaxime	271 (54%)	77 (15%)
Cefoperazone	288 (57.5%)	187 (37%)
Cefepime	454 (91%)	45 (9.0%)
Ceftriaxone	125 (25%)	13 (2.6%)
Ciprofloxacin	119 (24%)	358 (71.5%)
Chloramphenicol	367 (73.3%)	93 (18.6%)
Co-amoxiclav	326 (65%)	147 (29.3%)
Co-trimoxazole	211(42%)	238 (47.5%)
Colistin	16 (3.2%)	446 (89%)
Ertapenem	0 (0%)	29 (5.8%)
Gentamicin	7 (1.4%)	338 (67.5%)
Imipenem	3 (0.6%)	466 (93%)
Meropenem	0 (0%)	380 (75.8%)
Polymyxin	13 (2.6%)	468 (93.4%)
Piperacillin/tazobactam	311 (62%)	181 (36%)
Tigecycline	74 (14.8%)	393 (78.4%)
Teicoplanin	0 (0%)	1 (0.2%)
Erythromycin	0 (0%)	0 (0%)

Table 3 illustrates the drug resistance profiles of patients undergoing treatment for typhoid fever, highlighting significant insights into resistance patterns. MDR, defined as resistance to more than one antibiotic, was identified in 36% of patients (181 out of 501), while the majority (64% or 320 patients) did not exhibit MDR. This indicates that a substantial proportion of patients face challenges associated with MDR, complicating treatment options. In contrast, XDR, which refers to resistance to nearly all available antibiotics, was found in only 0.6% of patients (three out of 501), with 99.4% (498 patients) showing no evidence of XDR. Similarly, resistance to carbapenems, considered the last line of defense in antibiotic therapy, was exceedingly rare, affecting only 0.6% of patients (three out of 501), while 99.4% (498 patients) exhibited no resistance to carbapenems. Furthermore, gender and age did not show a significant impact on the patterns of drug resistance and sensitivity.

**Table 3:** Drug resistance profile of typhoid treatment patients

Drug Resistance	Yes	No
Multiple drug resistance	181 (36%)	320 (64%)
Extensive drug resistance	3 (0.6%)	498 (99.4%)
Resistance to carbapenems	3 (0.6%)	498 (99.4%)

Table 4 presents data on carbapenem resistance, MDR, and XDR bacteria in different genders and age groups. The prevalence of carbapenem resistance was found to be two (0.7%) in males and one (0.5%) in females, with a p-value of 0.644, indicating no significant difference between genders. Similarly, the prevalence of MDR was 106 (35.5%) in males and 75 (37.1%) in females, with a p-value of 0.384, suggesting no significant gender-based difference. For XDR, no males (0.0%) had XDR, while three (1.5%) females were affected, with p=0.065, indicating a borderline but not statistically significant difference. In terms of age, carbapenem resistance was observed in one (0.9%) of the 11-20-year group and two (0.6%) of the 21-30-year group, with p = 0.956, showing no significant age-related difference. MDR prevalence was 41 (36.0%) in the 11-20-year group, 129 (35.8%) in the 21-30-year group, eight (44.4%) in the 31-40-year group, and three (33.3%) in the 41-50-year group (p = 0.9), indicating no significant difference across age groups. XDR was present in one (0.9%) of the 11-20-year group and two (0.6%) of the 21-30-year group, with no cases in other age groups. The p-value of 0.956 indicates no significant difference in XDR across age categories. Overall, there were no significant gender or age differences in the prevalence of carbapenem resistance, MDR, or XDR.

**Table 4:** MDR, XDR, and carbapenems vs. gender and age Chi-square test MDR: multidrug-resistant; XDR: extensively drug-resistant

Category	Group	Carbapenems	p-value	MDR	p-value	XDR	p-value
Gender	Male	2 (0.7%)	0.644	106 (35.5%)	0.384	0 (0.0%)	0.065
	Female	1 (0.5%)		75 (37.1%)		3 (1.5%)	
Age	11–20 years	1 (0.9%)	0.956	41 (36.0%)	0.9	1 (0.9%)	0.956
	21–30 years	2 (0.6%)		129 (35.8%)		2 (0.6%)	
	31–40 years	0 (0.0%)		8 (44.4%)		0 (0.0%)	
	41–50 years	0 (0.0%)		3 (33.3%)		0 (0.0%)	

**Figure 1:**
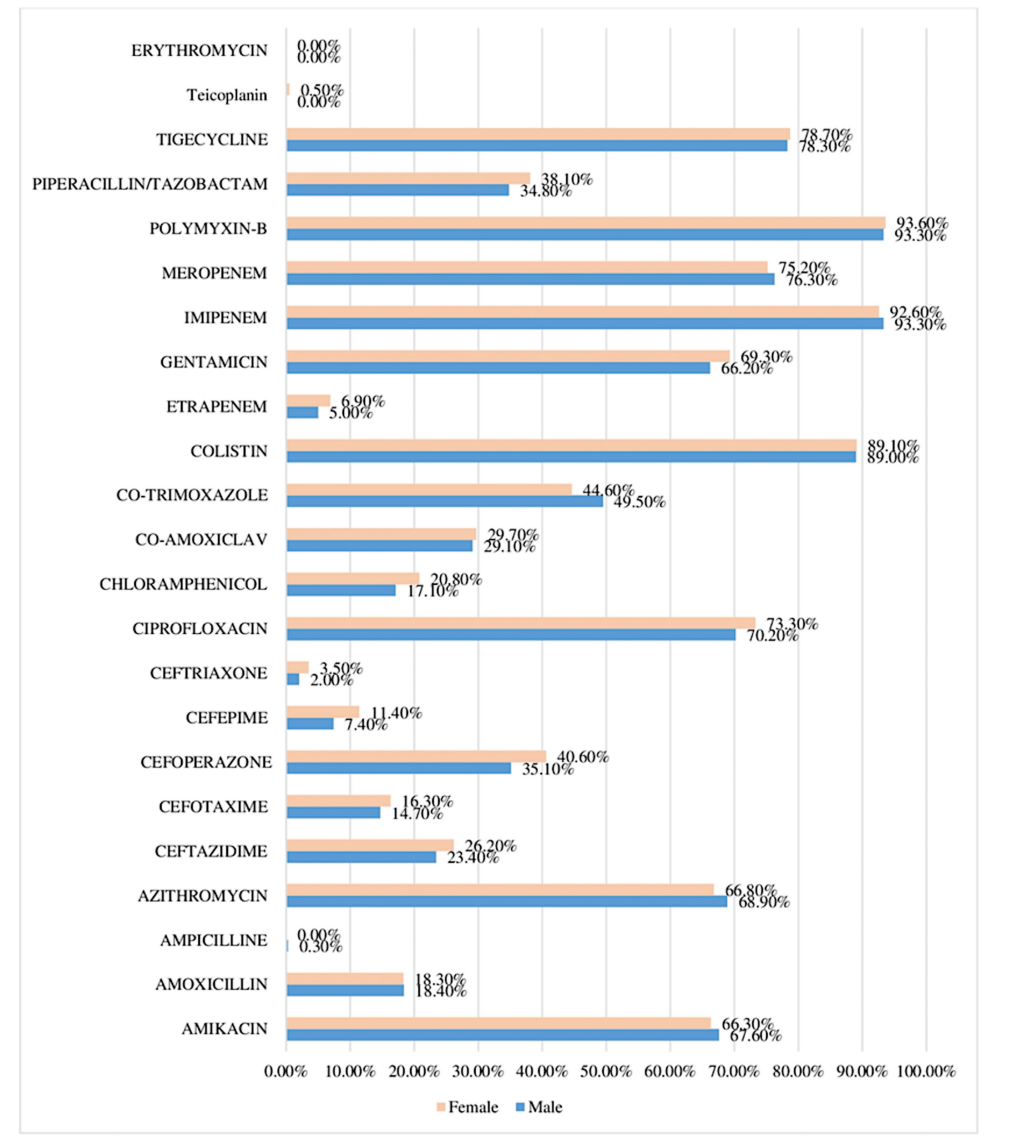
Gender vs. antibiotic sensitivity Image created by the authors.

Figure 1 illustrates the antibiotic sensitivity for males and females, with some variations across different antibiotics. Overall, sensitivity to imipenem, colistin, and polymyxin-B is very high for both genders, with nearly identical rates (approximately 93% and 89%, respectively). Ciprofloxacin and tigecycline also show high sensitivity, with females slightly surpassing males in both cases. There are minor gender differences in several antibiotics, with females showing slightly higher sensitivity to cefoperazone, cefepime, and chloramphenicol, while males show a slightly higher sensitivity to co-trimoxazole. For some antibiotics, such as amoxicillin and ampicillin, the sensitivity rates are almost identical between genders. Overall, the data show that gender has a minimal impact on antibiotic sensitivity, with most antibiotics exhibiting similar sensitivity across both males and females.

## Discussion

Our study provides a comprehensive analysis of the efficacy of carbapenems and other antibiotics in the treatment of typhoid fever, particularly concerning MDR and XDR strains of *Salmonella* Typhi at Hayatabad Medical Complex in Peshawar. The results reveal that imipenem exhibits an impressive sensitivity of 466 (93%) with only three (0.6%) resistance, while meropenem shows 380 (75.8%) sensitivity and no observed resistance, indicating their critical role as treatment options for XDR strains. These findings are consistent with broader resistance patterns reported in the literature; for example, Yousaf et al. (2024) reported a mere 3% resistance to meropenem, affirming its viability as a treatment option [17], while Al Masum et al. (2024) found 100% sensitivity to carbapenems in severe cases of *Salmonella* Typhi infection [11]. The rising prevalence of XDR *Salmonella* Typhi, reported to be as high as 43.4% in some studies [10], underscores the urgent necessity to preserve the efficacy of carbapenems through robust antimicrobial stewardship. This study highlights that carbapenems represent one of the last lines of defense against resistant typhoid strains, particularly as resistance to other antibiotics such as fluoroquinolones and third-generation cephalosporins escalates. Therefore, continuous surveillance and judicious use of carbapenems are essential to mitigate further resistance.

Despite their effectiveness, concerns arise regarding the growing reliance on carbapenems, as evidenced by the three (0.6%) resistance rates observed in our study, which pose significant challenges given the limited treatment options for XDR typhoid. Although this percentage is low, it emphasizes the critical need for careful use of carbapenems to prevent further resistance development, a concern corroborated by previous studies [18].

Our study also reveals significant sensitivity in non-carbapenem antibiotics, particularly polymyxins and colistin, which demonstrated superior efficacy against *Salmonella* Typhi, even surpassing that of carbapenems. Specifically, polymyxin showed a remarkable sensitivity of 468 (93.4%) with only 13 (2.6%) resistance, while colistin exhibited 446 (89%) sensitivity and 16 (3.2%) resistance. These results suggest that these non-carbapenem agents could serve as viable alternatives, helping to reduce reliance on carbapenems and preserve their effectiveness for more severe cases.

Furthermore, recent studies have noted a 100% sensitivity of *Salmonella* Typhi to imipenem and meropenem, alongside notable sensitivity to colistin and polymyxin E, especially when combined with adjuvants such as berberine and EDTA (ethylenediaminetetraacetic acid), which enhance colistin's efficacy against resistant strains [19,20]. However, the emergence of resistance mechanisms complicates treatment strategies; for example, research indicates that β-lactam antibiotics can inadvertently promote polymyxin tolerance by inducing outer membrane vesicle (OMV) production, which sequesters polymyxins and diminishes their effectiveness [21].

While polymyxin and colistin present promising alternatives, their association with significant nephrotoxicity limits their use, particularly in populations with underlying kidney conditions, necessitating careful monitoring to minimize adverse effects. Further research is required to explore the safety and efficacy of these drugs across diverse patient populations, particularly in resource-limited settings where healthcare access may be restricted.

The increasing prevalence of MDR and XDR *Salmonella* Typhi strains poses alarming resistance patterns in typhoid treatment, presenting substantial challenges for effective management globally. In Peshawar, 414 (82.6%) of *Salmonella* Typhi isolates were classified as XDR, with 59 (11.9%) identified as MDR [6]. A similar pattern was observed in Zanzibar, where 98% of isolates were MDR, with 69% exhibiting low-level ciprofloxacin resistance [22]. The mechanisms underlying this resistance are attributed to genomic mutations and horizontal gene transfer, complicating treatment options [23]. Genomic analyses have identified multiple resistance genes, including *blaTEM-1B* and *dfrA7*, prevalent in XDR strains [24]. A meta-analysis encompassing 13,000 *Salmonella* Typhi genomes highlighted that high-level ciprofloxacin resistance is particularly common in South Asia, with XDR strains predominant in Pakistan [25]. Despite these concerning trends, ongoing genomic surveillance and vaccination efforts are imperative to curtail the spread of resistant strains and enhance treatment outcomes.

In light of these findings, our research highlights significant resistance profiles that elucidate the challenges posed by antibiotic resistance in typhoid fever. The high resistance rates observed for amoxicillin (361, 72%) and co-amoxiclav (326, 65%) indicate their diminished effectiveness against *Salmonella* Typhi infections. Additionally, cephalosporins, such as cefepime and ceftazidime, displayed alarming resistance rates of 454 (91%) and 335 (67%), respectively, underscoring the urgent necessity for alternative treatment strategies. Interestingly, azithromycin exhibited moderate effectiveness, with a sensitivity of 341 (68%) and a resistance rate of 134 (26.7%). While azithromycin may still be a viable option for certain patients, rising resistance rates necessitate caution in its continued use as a first-line therapy, particularly in regions grappling with high antibiotic resistance. Notably, the low prevalence of XDR strains (three, 0.6%) in our study suggests that while they remain a significant threat, MDR typhoid is more prevalent, affecting 36% of patients.

Our study highlights the critical need for antimicrobial stewardship and increased vaccination efforts in endemic regions to prevent the emergence of carbapenem-resistant *Salmonella* Typhi, reduce the incidence of typhoid fever, and enhance public health through improved hygiene practices. The limitations of our study include its retrospective nature, relying solely on existing medical records from a single medical facility.

## Conclusions

This study highlights the critical role of carbapenems in the treatment of MDR and XDR typhoid fever at Hayatabad Medical Complex, Peshawar. Our findings indicate that while carbapenems, particularly imipenem and meropenem, exhibit high sensitivity rates of 466 (93%) and 380 (75.8%), respectively, and low resistance (three, 0.6%), non-carbapenem antibiotics such as polymyxin and colistin present even higher efficacy, with sensitivities of 468 (93.4%) and 446 (89%), respectively. Despite a relatively low prevalence of XDR cases (three, 0.6%) and resistance to carbapenems, the significant occurrence of MDR (181, 36%) underscores the urgent need for effective antimicrobial stewardship to preserve the efficacy of these critical treatment options. The study emphasizes the importance of continuous surveillance of antibiotic resistance patterns and the exploration of alternative therapeutic strategies to manage typhoid fever effectively.

In conclusion, while carbapenems remain a vital line of defense against resistant strains of *Salmonella* Typhi, reliance on these antibiotics must be balanced with efforts to promote responsible use of all antimicrobials in order to mitigate the risks associated with rising resistance and ensure effective treatment for future patients.
